# Supplementary material for: Determinants of hypertension among diabetes patients attending selected comprehensive specialized hospitals of the Amhara Region, Ethiopia: An unmatched case-control study
Source: PLoS One. 2022 Dec 16;17(12):e0279245. doi: 10.1371/journal.pone.0279245 (PMC9757598; doi:10.1371/journal.pone.0279245)
Supplement: Attachment — Submitted filename: manuscript for PLOS ONE.docx [file pone.0279245.s002.docx]

**Determinants of Hypertension among adult Diabetes Mellitus patients in Amhara Region comprehensive specialized hospitals, Amhara Region, Ethiopia, 2020/21. A multi-center Unmatched Case-Control Study**

Makda Abate Belew*^1^, Teshager Woldegiorgis Abate^2^, Alemshet Yirga Berhie^3^, Eleni Dagnaw Abeje^4^, Dawit Algaw Ayele ^5^, Melsew Dagne Abate^6^, Rediet Akele Getu^7^, Berihun Bantie^8^, Sewnet Getaye Workie ^9^

**^1^** Department of Nursing, school of nursing and midwifery, College of medicine and health science, Debre Berhan University, Debre Berhan, Ethiopia; **^2^** Department of Adult Health Nursing, College of Medicine and Health Science, Bahir Dar University, Bahir Dar**,** Ethiopia; ^3^ Department of Adult Health Nursing, College of Medicine and Health Science, Bahir Dar University, Bahir Dar**,** Ethiopia; **^4^** Department of nursing, School of nursing and midwifery, college of medicine and health science, Debre Berhan University, Debre Berhan, Ethiopia; **^5^** Department of nursing, Bahir Dar health science Collage, Bahir Dar**,** Ethiopia; **^6^** Department of Nursing, College of Health Sciences, Woldia University, Woldia, Ethiopia. ^7^ Department of Nursing, school of nursing and midwifery, College of medicine and health science, Debre Berhan University, Debre Berhan, Ethiopia; ^8^Department of Nursing, College of medicine and health science, Debre Tabor University, Debre Tabor, Etiopia; ^9^Department of public health, school of public health, college of medicine and health science, Debre Berhan University, Debre Berhan, Ethiopia.

**Correspondence:** Makda Abate Belew, **Email:** [sismakda@gmail.com](mailto:sismakda@gmail.com)

# Abstract

**Background:** Comorbidity of hypertension with diabetes mellitus is a worldwide public health problem causing significant morbidity and mortality, and decreases quality of live. Despite the increasing burden of hypertension among DM patients, there isn’t enough data on determinants of hypertension among DM patients in Amhara region, Ethiopia. Hence, this study aimed to identify determinants of hypertension among people with diabetes attending chronic follow-up clinics in Amhara region comprehensive specialized hospitals, Ethiopia, 2021.

**Method and Materials:** an institutional-based unmatched case-control study was conducted among 476 participants in Amhara region comprehensive specialized hospitals (Debre Berhan, Felege Hiwot, and Dessie Comprehensive specialized hospital). A multistage sampling technique was used to select participants of this study. Data were collected by using interviewer-administered questionnaires, physical measurements, and data extraction checklist. Logistic regression was used to identify variables having significant association at p-value < 0.05 with a 95% confidence interval.

**Results:** A total of 235 cases and 235 controls participated in this study. The mean age ± Standard deviation for the controls was 60.12 ± 10.387 and the median age for the case was 60 with an interquartile range of (12- 66). The significant determinants of hypertension with AOR [95% CI] were age > 50 years, a lower level of physical activity: 1.82 [1.00, 3.31], depression: 2.00 [1.24, 3.21], family history of hypertension: 2.13 [1.34, 3.37], not attending a diabetic education session: 1.87 [1.18, 2.96], longer duration of diabetes: 1.99 [1.05, 3.79] and poor glycemic control: 1.57 [1.01, 2.45].

**Conclusion:** This finding suggests that as an integral part of diabetes care, health care providers should screen, assess and treat depression, create participants' awareness to attend diabetes education, and advise diabetes individuals to engage in physical activity.

# Introduction

Diabetes mellitus (DM) is a serious, chronic metabolic condition characterized by a state of hyperglycemia resulting from defects in insulin secretion, insulin action, or both. According to the 2021 estimation of the international diabetes federation (IDF) around 531 million adults aged from 20 to 79 years old (10.5% of all adults in this age group) are living with diabetes. This figure is predicted to rise to 643 million by 2030 and 783 million by 2045 (1). Among fourty-eight Sub-Saharan African countries and territories in the IDF Africa Region, Ethiopia took fourth place with the number of people living with DM in 2021 (1), and according to a recent systematic review and meta-analysis, the pooled prevalence of DM was 4.99% (2). Over the globe, around 6.7 million adults (20–79) were estimated to die due to diabetes or its complications including hypertension in 2021(1). Approximately one-third (32.6%) of all deaths from diabetes occurs in people of working age (under the age of 60) (1).

Hypertension (HTN) among people with diabetes mellitus is a serious medical condition characterized by persistent elevation of systemic arterial blood and clinically defined as systolic blood pressure (SBP) of >130 mmHg and/or diastolic Blood pressure (DBP) of >80 mmHg on two consecutive days or any prior diagnosis of HTN made by health personnel and taking antihypertensive drugs (3, 4). The frequency of HTN among the diabetes population is almost twice that of non-diabetic populations and it is reported in over two-thirds of people with type 2 diabetes (5). The highest percentage of hypertension among diabetic clients is attributed to different structural alterations in the microcirculation secondary to endothelial dysfunction, inflammation, oxidative stress, insulin resistance in the nitric-oxide pathway, dyslipidemia, smooth muscle growth, sodium fluid retention, the stimulatory effect of hyperinsulinemia on the sympathetic nervous activity, and the excitatory effect of hyperglycemia on renin-angiotensin-aldosterone system (1, 5-7).

The coexistence of hypertension with DM increases the risk of cardiovascular events by six-fold, double the risk of all-cause mortality and stroke, triple the risk of coronary heart diseases, hasten the progression of both microvascular and macrovascular complications, reduces the event-free survival, and influences the ability to achieve the recommended therapeutic targets, both in terms of fasting glucose and blood pressure (BP) levels (8). One study showed that the coexistence of hypertension with diabetes mellitus is attributed to the risk of death and cardiovascular events by 44% and 41%, respectively, in comparison with 7% and 9% of risks in people with diabetes alone (9). If the level of blood pressure isn’t controlled, it leads to heart attack, stroke, kidney disease, vision loss, sexual dysfunction, and peripheral arterial disease (5). Moreover, the development of HTN in diabetic individuals complicates the treatment strategy, considerably impairs health-related quality of life (HRQoL), and cause a huge economic impact as a result of direct costs of treatment and human power lost due to the devastating effect of the diseases on the individual, family, and society as a whole (6, 10, 11).

Behavioral risk reduction (cessation of tobacco smoking, following a healthy diet plan, doing physical activity, and minimizing alcohol consumption) and lifestyle modification (weight loss if overweight or obese, a DASH style based nutrition counseling, and reduced-sodium/salt intake) were some of the strategies which have been implemented in the past to control the progress of hypertension among people with DM (12). Despite the implementation of those strategies, different studies indicated that the burden of hypertension among people with diabetes is still high as they indicate its prevalence is 76% in Jordan (13), 92.7% in Malaysia (14), 56.3% in Nigeria (15), 85.6% in Benghazi (16), 56.3% in Adama (17) and 59.5% in Debre Tabor general hospital (6).

Even though Ethiopia is among the top five leading African countries with the prevalence of DM, there is still a paucity of studies conducted on the determinants of hypertension among people with DM in Ethiopia. A case-control study conducted in Tigray Ethiopia on determinants of hypertension among people with DM (18) recommended conducting further research that investigates the determinants of hypertension among people with diabetes in a broader social context and larger sample size. This research tried to fill this gap by broadening the study area, relatively increasing the sample size, and adding some more variables.

Additionally, in view of findings of different institutional-based cross-sectional studies (6, 11, 17-19), the present study aims at filling the existing research gap by examining multiple risk factors such as impaired physical activity, unhealthy diet, kchat chewing, depression, poor social support, harmful alcohol consumption, overweight, obesity, raised waist to hip ratio and raised waist circumference which may contribute to the progress of hypertension among people with DM.

# Methods and materials

## Study setting, design, and period

An institutional**-**based unmatched case-control study was conducted from March 17, 2021, to April 18, 2021, among people with diabetes mellitus in the Amhara region's comprehensive specialized hospitals. There are eight comprehensive specialized hospitals in the region and an estimated total number of DM clients attending their follow-up in those eight hospitals is approximately 18,573.

Debre-Berhan, Felege-Hiwot, and Dessie comprehensive specialized hospitals were included in this study out of the eight comprehensive specialized hospitals. The chronic diseases follow-up clinics of those hospitals give services on all working days (Monday to Friday) and are approximately visited by twenty-five to thirty DM clients per day.

## Population

All adult people with diabetes mellitus attending chronic follow-up clinics in Amhara region comprehensive specialized hospitals were the source population. Adult people with diabetes mellitus who have hypertension and attending the chronic disease follow-up clinic in the selected hospitals during the study period were considered as cases. Whereas, Adult people with diabetes mellitus who have no hypertension and attended the chronic disease follow-up clinic in the selected hospitals during the study period were controls. Critically ill DM patients or those who have a severe medical illness that made it impossible to complete the study questionnaire in one setting, newly diagnosed diabetic patients who didn’t take diabetes medication within the last 6 months in a regular follow up, recently transferred in diabetes mellitus patients who have incomplete medical records, those who have a history of hypertension at the time of diagnosis of DM and before the diagnosis of DM were excluded from this study.

## Sample size determination and sampling procedure

Epi info version 7.2.3.1 was used to calculate sample size by assuming 95% Confidence interval (CI), 80% power, and 1:1 case to controls ratio. Odds ratio (OR) and proportion of potential predictor variable of hypertension among control were taken from a study in Tigray, Ethiopia (18). By considering adjustment for expected non-response rate (10 %) and design effect (1.5), the largest total sample size became 476.

A multistage sampling technique was used to select participants of this study. Initially, a list of all comprehensive specialized hospitals was obtained from the Amhara health bureau. There are eight comprehensive specialized hospitals in the Amhara region. Of all eight comprehensive specialized hospitals, three comprehensive specialized hospitals (Debre Berhan, Dessie, and Felegehiwot) were selected by using a simple random sampling method. After that, the number of diabetic patients with hypertension and without hypertension who took service from chronic follow-up clinics were taken from the chronic disease follow-up clinic registry book. Finally, eligible cases were selected every two patients by using systematic random sampling. For each case, one control that fulfills the inclusion criteria was selected every seven patients by using a systematic random sampling technique from chronic follow-up clinics of the same hospitals from which cases were drawn.

## Study variables

The dependent variable of the current study was Hypertension among people with DM. Independent variables include Socio-demographic variables, clinically related variables, Psychosocial and Behavioral variables.

## Operational definition

**HTN Comorbid with DM:** diabetic individuals diagnosed by a physician and on antihypertensive medications or those who had systolic blood pressure 140 mmHg and/or diastolic blood pressure 90 mmHg (4).

**Adherence to physical activity:** patients were considered as having higher, moderate, or lower adherence to physical activity if they meet any of the criteria for higher, moderate, or lower levels of physical activity which is put on IPAQ screening protocol (20).

**Alcohol consumption**: - patients scoring 8 or more on the alcohol use disorder identification test (AUDIT) are considered as hazardous alcohol consumers (21, 22).

**Adherence to dietary regimen:** on the perceived dietary adherence questionnaire (PDAQ), patients were classified as having good dietary adherence if they eat a healthy diet for at least four days in the week (23).

**Medication adherence:** on Morisky Medication Adherence scale – 8 (MMAS-8) related questions patients were considered as having good adherence when they score 8, medium adherence if they score 6 to less than 8, and low adherence if score < 6 (24).

**Glycemic control:** the patient was considered as having good glycemic control if the average of the last three fasting blood glucose level is between 70mg/dL to 130mg/dL or Poor glycemic control if the average of the last three fasting blood glucose level >130 mg/dL (25).

**Adherence to self-blood glucose monitoring:** the patient was considered adhere to blood glucose monitoring when he/she score at least 50% self-blood glucose monitoring-related questions (26).

**Body Mass Index**: Patients were considered as Underweight if BMI is less than 18.5 Kg/m2, healthy weight if BMI is 18.5 to 24.9 Kg/m2, overweight if BMI is from 25 to 29.9 Kg/m2, and obese if BMI is 30Kg/m2 or higher.

**Social support:** on Oslo Social Support questionnaires 3 (OSS-3) a patient with a score of 3 - 8 was considered as having “poor social support”, 9–11 as “moderate social support” and 12–14 as “strong social support” (27).

**Depression:** patients with a score of 5 and above on the Patient Health Questionnaire 9(PHQ-9) were considered as having depression. On PHQ-9 depression subscales patients were classified as having no depression if the PHQ-9 score is 0–4, mild depression if the PHQ-9 score is 5–9, moderate depression if the PHQ-9 score is 10–14, moderately severe depression if the PHQ-9 score is 15 –19, and severe depression if the PHQ-9 score is 20–27 (25).

**Waist circumference**: >94 cm for male and >80 cm for female was considered as abnormal(raised/above normal) (28).

**Waist to hip ratio**: ≥ 0.90 for males and ≥ 0.85 cm for females was considered as abnormal (raised/above normal) (28).

## Data collection tools and procedures

Data were collected by using semi-structured interviewer-administered questionnaires, physical measurement, and data extraction checklists which developed after reviewing different works of literature (6, 11, 16-19, 29, 30). Data was collected by six BSC nurses and supervised by three MSc students. The questionnaire contains different parts which are socio-demographic data, clinical characteristics of the study subjects, behavioral and psychosocial characteristics, physical measurements, and a data extraction checklist.

Cleints level of physical activity was assessed using short-form of international physical activity questionnaire (IPAQ) (31). This tool has been used in previous studies conducted in Ethiopia (32, 33).

MMAS - 8 (24, 34) was used to measure the patient's Self-reported adherence to diabetic medication.

PDAQ, a nine-item tool is also used for assessing dietary adherence (23). PHQ-9, a nine-item self-report instrument (scoring ranges from 0 to 27), and a standardized validated tool in East Africa including Ethiopia was used for assessing depression (35). The level of social support was assessed using Oslo 3 social support scale by asking the patients to rate the level of support they received from family and friends. This tool is validated in different African countries (36) and has been used in studies conducted in Ethiopia (27). The 10-item AUDIT tool was used to assess alcohol consumption level (3 items), symptoms of alcohol dependence (3 items), and problems associated with alcohol use (4 items) (21, 22). Patients were considered hazardous alcohol consumers if they scored above 8. Recent clinical related and biochemical data like fasting blood glucose level, presence of co-morbidity, diabetes-related complications, duration of diabetes, and treatment modality were collected from the patients’ records by using checklists.

**Physical Measurements**

After the participants stood with arms at the sides, feet positioned close together, and weight evenly distributed across the feet, waist circumference was measured to the nearest 0.1cm at the end of normal exhalation at the level of the iliac crest. The hip circumference of the patients was measured to the nearest centimeter at the largest maximum circumference of the buttocks. Both hip and waist circumference were measured with stretch-resistant tape wrapping snugly around the participants. WHR was calculated as WC (cm) divided by HC (cm) (28).

Heights of subjects were measured to the nearest centimeter, using a standard meter with subjects standing in the upright position. Weight (in kilograms) was measured in light clothing using a calibrated Weighing Scale and recorded to the nearest 0.1 kg. BMI was calculated as the weight divided by height squared (kg/m2) (28). The scale was calibrated regularly and the indicator was checked against zero reading before every measurement.

## Data processing and analysis

Data were first checked for completeness and then each completed questionnaire was assigned a unique code. Epi Data version 3.1 and Statistical Package for Social Sciences (SPSS) version 21 were used for data entry and analysis respectively. The data was checked by visualizing, calculating frequencies, and sorting. Based on the nature of variables frequency distribution, summary statistics such as mean, median, standard deviation, and interquartile range were computed for case and control groups after doing a normality test (for continuous variables). The bivariable logistic regression model was fitted for each explanatory variable. Accordingly, those variables having a p-value less than or equal to 0.2 in the bivariable analysis were taken as candidates for the multivariable logistic regression model. In a multivariable regression P-value, less than 0.05 with a 95% confidence interval was considered as statistically significant. The fitness of the model was checked using Hosmer and Lemeshow goodness-of-fit test statistics giving a p-value of 0.463.

Ethical consideration

Ethical clearance was obtained from the institutional review board of Bahir Dar University, college of medicine, and health sciences with a protocol number 079/2021. A formal letter was submitted to the selected hospitals. Initial to data collection, informed consent was obtained from respondents, and confidentiality of response was maintained throughout the study. Patients found to have severe depression were linked to psychiatry clinics for further assessment and treatment.

# Results

**Socio-demographic characteristics** **of the study participants**

A total of 470 diabetic individuals (235 cases and 235 controls) were included in this study yielding a response rate of 98.74%. Of the total respondents, 121(51.5%) of controls and 118(50.2%) of cases were male participants. The mean age (± Standard deviation) for the controls was 60.12 (SD: ± 10.387) and the median age for the cases was 60 with an interquartile range of (66 -12 = 14). Occupation-wise, 57 (24.3%) of controls were government employees and 59 (25.1%) of cases were retired. Of all participants enrolled in this study, 105 (44.7%) of cases and 97 (41.3%) of controls had an average monthly income of >3000ETB (Table 1).

Table 1: Sociodemographic characteristics of study participants for determinants of hypertension among diabetes mellitus patients having follow-up in chronic disease follow-up clinics of Amhara region comprehensive specialized hospitals, Ethiopia, 2021.

| **Variables** | **Controls (n=235)** | | **Cases (n=235)** | |
| --- | --- | --- | --- | --- |
|  | **Frequency** | **%** | **Frequency** | **%** |
| Sex | | | | |
| Male | 121 | 51.5 | 118 | 50.2 |
| Female | 114 | 41.5 | 117 | 49.8 |
| Age |  |  |  |  |
| ≤ 50 | 123 | 52.3 | 45 | 19.2 |
| 51-60 | 59 | 25.1 | 76 | 32.3 |
| 61-70 | 37 | 15.7 | 76 | 32.3 |
| >70 | 16 | 6.9 | 38 | 16.2 |
| Marital status | | | | |
| Single | 34 | 14.4 | 12 | 5.1 |
| Married | 160 | 68.1 | 176 | 74.9 |
| Divorced | 23 | 9.8 | 17 | 7.2 |
| Widowed | 18 | 7.7 | 30 | 12.8 |
| Educational status of the respondents | | | | |
| Unable to Read and Write | 54 | 23.0 | 47 | 20.0 |
| Read and write | 31 | 13.2 | 35 | 14.9 |
| Primary school | 45 | 19.1 | 39 | 16.6 |
| Secondary school | 22 | 9.4 | 28 | 11.9 |
| Preparatory school | 29 | 12.3 | 39 | 16.6 |
| College or university completed | 54 | 23.0 | 47 | 20.0 |
| Occupational status | | | | |
| Government Employee | 57 | 24.3 | 49 | 20.9 |
| Retired | 34 | 14.4 | 59 | 25.1 |
| Housewife | 43 | 18.3 | 45 | 19.1 |
| Daily laborer | 16 | 6.8 | 9 | 3.8 |
| Merchant | 32 | 13.6 | 44 | 18.7 |
| Farmer | 53 | 22.6 | 29 | 12.4 |
| Residence | | | | |
| Urban | 170 | 72.3 | 196 | 83.4 |
| Rural | 65 | 27.7 | 39 | 16.6 |
| Average family income of the family | | | | |
| 500-1000 ETB | 38 | 16.2 | 22 | 9.4 |
| 1001-2000 ETB | 55 | 23.4 | 67 | 28.5 |
| 2001-3000 ETB | 45 | 19.1 | 41 | 17.4 |
| >3000 ETB | 97 | 41.3 | 105 | 44.7 |

**ETB means that Ethiopian Birr**

**Behavioral and psychosocial Characteristics** **of the study participants**

As the finding of this study shows majority of 190 (80.9%) cases and 187(79.6%) controls were not kchat chewers. According to this study, one-quarter of the cases (25.1%) and nearly one-third of controls (28.5%) were hazardous alcohol consumers. Slightly lower than half of the cases (47.2%) and two-fifths of controls (40%) were found to have lower medication adherence. Concerning adherence to diet more than half of the cases (54.9%) and more than three-fifths of the controls (64.5%) had good adherence to a diet. The majority of cases (79.6%) and controls (84.7%) did not adhere to SBGM. Additionally, more than one-quarter of controls (28.9%) and nearly half of cases (49.8%) reported a lower level of physical activity in the last seven days. Among the total participants enrolled in this study 166 (70.6%) cases and 122 (51.9%) of controls were found to have depression. Regarding social support from the total participants, 102 (43.4%) of cases and 106 (45.1%) of controls have received a moderate level of social support from their family, friends, and neighbors (Table 2).

Table 2:Psychosocial and behavioral characteristics of study participants for determinants of hypertension among diabetes mellitus patients having follow-up in chronic disease follow-up clinics of Amhara region comprehensive specialized hospitals, Ethiopia, 2021.

| **Variables** | **Cases (n=235)** | | **Controls (n=235)** | |
| --- | --- | --- | --- | --- |
|  | **Frequency** | **%** | **Frequency** | **%** |
| Have you ever chewed kchat | | | | |
| Yes | 45 | 19.1 | 48 | 20.4 |
| No | 190 | 80.9 | 187 | 79.62 |
| How often do you chew kchat | | | | |
| Less than once a month | 2 | 4.4 | 3 | 6.3 |
| Once to three times per month | 1 | 2.2 | 8 | 16.7 |
| Once a week | 12 | 26.7 | 16 | 33.3 |
| Two to four times per week | 16 | 35.6 | 13 | 27.1 |
| Daily | 14 | 31.1 | 8 | 16.6 |
| Hazardous alcohol consumption | | | | |
| Yes | 59 | 25.1 | 67 | 28.5 |
| No | 176 | 74.9 | 168 | 71.5 |
| Alcohol dependency | | | | |
| Low Risk | 176 | 74.9 | 168 | 71.5 |
| Medium Risk | 32 | 13.6 | 43 | 18.3 |
| High Risk | 9 | 3.8 | 10 | 4.3 |
| Addiction Likely | 18 | 7.7 | 14 | 6.0 |
| Medication adherence | | | | |
| Low | 111 | 47.2 | 94 | 40.0 |
| Medium | 75 | 31.9 | 79 | 33.6 |
| Good | 49 | 20.9 | 62 | 26.4 |
| Dietary adherence | | | | |
| Good | 151 | 64.3 | 129 | 54.9 |
| Poor | 84 | 35.7 | 106 | 45. |
| Adherence to self-blood glucose monitoring | | | | |
| Adhere | 36 | 15.3 | 48 | 20.4 |
| Not adhere | 199 | 84.7 | 187 | 79.6 |
| Level of physical activity | | | | |
| Low | 118 | 50.2 | 68 | 28.9 |
| Moderate | 75 | 31.9 | 100 | 42.6 |
| High | 42 | 17.9 | 67 | 28.5 |
| Depression | | | | |
| Yes | 166 | 70.6 | 122 | 51.9 |
| No | 69 | 29.4 | 113 | 48.1 |
| Severity of depression | | | | |
| No | 69 | 29.4 | 113 | 48.1 |
| Mild | 78 | 33.2 | 64 | 27.2 |
| Moderate | 56 | 23.8 | 31 | 13.2 |
| Moderately sever | 25 | 10.6 | 20 | 8.5 |
| Sever | 7 | 3.0 | 7 | 3.0 |
| Depression related level of difficulty in doing work | | | | |
| Not difficult at all | 155 | 66.0 | 133 | 56.6 |
| Somewhat difficult | 51 | 21.7 | 74 | 31.5 |
| Very difficult | 22 | 9.3 | 25 | 10.6 |
| Extremely difficult | 7 | 3.0 | 3 | 1.3 |
| Social Support | | | | |
| Poor | 79 | 33.6 | 67 | 28.5 |
| Moderate | 102 | 43.4 | 106 | 45.1 |
| Strong | 54 | 23.0 | 62 | 26.4 |

**Clinical characteristics of the study participants**

Regarding the type of DM in this study, 96.6% of cases and 81.7% of controls were found to have type-two diabetes. The median age of cases at diagnosis of DM was 52 with an interquartile range of (59 – 44 = 15) and the mean age of controls at diagnosis of DM was 44.49 ± (SD: 11.0). Concerning the type of treatment taken for DM, the largest percentage of cases (83%) and nearly three fourth of controls (71.9%) were on oral hypoglycemic agents. The median duration of diabetes mellitus since diagnosis was 8 with an interquartile of (12-5 =7) for both controls and cases**.** More than half of the cases 120 (51.1%) and more than one-quarter of the controls 66 (28.1%) reported that they had a family history of hypertension.

Concerning education about diabetes more than half of the cases 134(57%) and nearly three fourth of controls 170(72.3%) have received diabetes education. Regarding glycemic control, unlike the cases 126 (53.6%) most of the controls 153 (65.1%) had good glycemic control. According to this study out of 470 participants more than half of the controls (56.2%) and two-fifths of the cases (40%) have a healthy weight. Concerning waist circumference higher than half of the controls (51.5%) and more than three-fifths of cases (68.5%) had above normal waist circumference. Nearly half of the controls (47.7%) and less than three fourth of cases (68.1%) had above the normal waist to hip ratio (Table 3).

Table 3: Clinical characteristics of study participants for determinants of hypertension among diabetes mellitus patients having follow-up in chronic disease follow-up clinics of Amhara region comprehensive specialized hospital, Amhara region, Ethiopia, 2021.

| **Variables** | **Cases (n=235)** | | **Controls (n=235)** | | |
| --- | --- | --- | --- | --- | --- |
|  | **Frequency** | **%** | **Frequency** | | **%** |
| Type of DM | | | | | |
| Type 1 | 8 | 3.4 | 43 | | 18.3 |
| Type 2 | 227 | 96.6 | 192 | | 81.7 |
| Kind of treatment of DM | | | | | |
| Oral hypoglycemic agent | 195 | 83.0 | 169 | | 71.9 |
| Insulin therapy only | 25 | 10.6 | 58 | | 24.7 |
| Both oral and insulin | 15 | 6.4 | 8 | | 3.4 |
| Duration of diabetes | | | | | |
| <5year | 46 | 19.5 | 85 | | 36.2 |
| 5-10year | 108 | 46.0 | 102 | | 43.4 |
| >10year | 81 | 34.5 | 48 | | 20.4 |
| Family history of HTN | | | | | |
| Yes | 120 | 51.1 | 66 | | 28.1 |
| No | 115 | 48.9 | 169 | | 71.9 |
| Family history of DM | | | | |  |
| Yes | 105 | 44.7 | 111 | | 47.2 |
| No | 130 | 55.3 | 124 | | 52.8 |
| Regularly visit a physician | | | | | |
| Yes | 206 | 87.7 | 195 | | 83.0 |
| No | 29 | 12.3 | 40 | | 17.0 |
| Diabetic health education | | | | | |
| Yes | 134 | 57.0 | 170 | | 72.3 |
| No  Comorbidity other than HTN  Yes | 101  110 | 43.0  46.8 | 65  55 | | 27.7  23.4 |
| No | 125 | 53.2 | 180 | | 76.6 |
| Specific types of comorbidities * | | | | | |
| Cardio vascular disease | 79 | 71.8 | 42 | | 76.4 |
| Respiratory disease | 17 | 15.5 | 4 | | 7.3 |
| Renal disease | 19 | 17.3 | 11 | | 20 |
| Neurological disease | 5 | 7.3 | 8 | | 9.1 |
| Diabetes-related complication | | | | | |
| Yes | 57 | 24.3 | 35 | 14.9 | |
| No | 178 | 75.7 | 200 | 85.1 | |
| Type of diabetes-related complication * | | | | | |
| Diabetic retinopathy | 5 | 14.3 | 9 | 15.8 | |
| Diabetic nephropathy | 12 | 34.3 | 26 | 45.6 | |
| Diabetic neuropathy | 13 | 37.1 | 19 | 33.3 | |
| Sexual dysfunction | 7 | 20.0 | 3 | 5.3 | |
| Average fasting blood Glucose | | | | | |
| Good | 126 | 53.6 | 153 | | 65.1 |
| Poor | 109 | 46.4 | 82 | | 34.9 |
| Body mass index | | | | | |
| Underweight | 1 | 0.4 | 6 | | 2.5 |
| Healthy weight | 94 | 40.0 | 132 | | 56.2 |
| Overweight | 105 | 44.7 | 70 | | 29.8 |
| Obese | 35 | 14.9 | 27 | | 11.5 |
| Waist Circumference | | | | | |
| Normal | 74 | 31.5 | 114 | | 48.5 |
| Above normal | 161 | 68.5 | 121 | | 51.5 |
| Waist to Hip Ratio | | | | | |
| Normal | 75 | 31.9 | 123 | | 52.3 |
| Above Normal | 160 | 68.1 | 112 | | 47.7 |

***Indicates that** **total will not add up to 235 (100%) for cases and 235 (100%) for control, as multiple responses were possible in these categories**

**Determinants of hypertension among DM patients**

On the bivariable logistic regression analysis respondent's age, marital status, educational status, average family income, non-adherence to medication, lower level of physical activity, non-adherence to diet, depression, body mass index, waist circumference, waist to hip ratio, family history of hypertension, not having diabetic health education, longer duration of diabetes, comorbidity other than hypertension., diabetes-related complication and average fasting blood glucose shows a statistically significant association at p-value < 0.2.

After controlling the potential confounders age, physical inactivity, depression, family history of hypertension, not attending a diabetic health education, duration of diabetes, and poor glycemic control were significant factors that made association at p-value < 0.05.

In this study diabetic patients who belongs within the age group of 51- 60 years were 3.33 times [AOR=3.331, 95 % CI (1.92 – 5.78)], 61-70 years were 3.99 times [AOR= 3.99, 95 % CI (2.14 – 7.46)] and >70 years were 2.95 times [AOR = 2.95, 95 % CI (1.25, 5.98)] more likely to have hypertension as compared to those diabetic individuals who aged below 50 years.

The odds of having hypertension were also 1.82 times [AOR = 1.82, 95 % CI (1.00 – 3.31)] higher among diabetic individuals who had a lower level of physical activity as compared to those who have higher levels of physical activity. Additionally, diabetic individuals who had depression were also 2 times [AOR = 2.00, 95 % CI (1.24 - 3.21)] more likely to be hypertensive than those diabetic individuals who have no depression.

Regarding family history, diabetic individuals who have a positive family history of hypertension were 2.13 times higher odds of [AOR = 2.13, 95 % CI (1.34 - 3.37)] having hypertension compared to those who had no family history of hypertension. Diabetic clients who didn’t attend diabetic education were 1.87 times [AOR= 1.87, 95% CI (1.18 – 2.96)] more likely to have hypertension than their counterparts.

The odds of having hypertension were also 1.99 times higher among those individuals who spent >10years of their life with DM as compared to those individuals spending their time with DM for less than 5 years [AOR= 1.99, 95% CI (1.05 – 3.79)]. Concerning glycemic control, diabetic individuals who have poor glycemic control were 1.57 times more likely to develop hypertension than those diabetic clients with good glycemic control [AOR=1.57, 95% CI (1.01 – 2.45)] (Table 4).

Table 4: Bivariable and Multivariable logistic regression analysis results for determinants of hypertension among DM patients having follow-up in chronic follow-up clinics of Amhara region comprehensive specialized hospital, Amhara region, Ethiopia, 2021.

| **Variables** | **Cases**  **(n = 235)** | **Controls**  **(n = 235)** | **COR**  **(95%CI)** | **AOR**  **(95%CI)** | **P**  **Value** |
| --- | --- | --- | --- | --- | --- |
| Age | | | | | |
| >70 | 38 (16.2) | 16 (6.8) | 6.492(3.30, 12.77) | 2.95 (1.25, 6.92) | 0.013** |
| 61-70 | 76 (32.3) | 37 (15.7) | 5.61 (3.34, 9.45) | 3.99 (2.14, 7.46) | < 0.001** |
| 51-60 | 76 (32.3) | 59 (25.1) | 3.52 (2.17,5.70) | 3.33 (1.92, 5.78) | < 0.001** |
| ≤ 50 | 45 (19.2) | 123 (52.3) | 1 | 1 |  |
| Marital status | | | | | |
| Married | 176 (74.9) | 160 (68.1) | 3.11 (1.56, 6.23) | 1.56 (0.67, 3.64) | 0.305 |
| Divorced | 17 (7.2) | 23 (9.8) | 2.09 (0.84, 5.19) | 1.15 (0.39, 3.39) | 0.797 |
| Widowed | 30 (12.8) | 18 (7.7) | 4.72 (1.96, 11.39) | 1.10 (0.37, 3.32) | 0.865 |
| Single | 12 (5.1) | 34 (14.4) | 1 | 1 |  |
| Educational status | | | | | |
| Unable to Read and Write | 47 (20.0) | 54 (23.0) | 1.00 (0.58, 1.74) | 0.89 (0.41, 1.96) | 0.779 |
| Read and write | 35 (14.9) | 31 (13.2) | 1.29 (0.69, 2.4) | 1.05 (0.46, 2.36) | 0.915 |
| Primary school | 39 (16.6) | 45 (19.1) | 0.99 (0.55, 1.77) | 0.96 (0.45, 2.05) | 0.910 |
| secondary school | 28 (11.9) | 22 (9.4) | 1.46 (0.74, 2.89) | 1.16 (0.49, 2.74) | 0.744 |
| preparatory school | 39 (16.6) | 29 (12.3) | 1.54 (0.83, 2.87) | 1.13 (0.51, 2.48) | 0.767 |
| College or university completed | 47 (20.0) | 54 (23.0) | 1 | 1 |  |
| Average family income | | | | | |
| >3000 | 105 (44.7) | 97 (41.3) | 1.86 (1.03, 3.38) | 1.609 (0.72, 6.62) | 0.251 |
| 2001-3000 | 41 (17.4) | 45 (19.1) | 1.57 (0.80, 3.08) | 1.264 (0.54, 2.54) | 0.592 |
| 1001-2000 | 67 (28.5) | 55 (23.4) | 2.10 (1.11, 3.96) | 1.77 (0.81, 3.86) | 0.151 |
| 500-1000 | 22 (9.4) | 38 (16.2) | 1 | 1 |  |
| Medication adherence | | | | | |
| Low | 111 (47.2) | 94 (40) | 1.49 (0.93, 2.37) | 1.15 (0.64, 2.04) | 0.646 |
| Medium | 75 (31.9) | 79 (33.6) | 1.20 (0.73, 1.961) | 0.93 (0.51, 1.69) | 0.802 |
| Good | 49 (20.9) | 62 (26.4) | 1 | 1 |  |
| Physical activity | | | | | |
| Low | 117 (49.8) | 68 (28.9) | 2.74 (1.69, 4.47) | 1.82 (1.00, 3.31) | 0.049** |
| Moderate | 76 (32.3) | 100 (42.6) | 1.21 (0.75,1.97) | 1.02 (0.57, 1.84) | 0.947 |
| High | 42 (17.9) | 67 (28.5) | 1 | 1 |  |
| Dietary Adherence | | | | | |
| Poor | 84 (35.7) | 106 (45.1) | 0.67 (0.47, 0.98) | 0.75 (0.47, 1.19) | 0.219 |
| Good | 151 (64.3) | 129 (54.9) | 1 | 1 |  |
| Depression | | | | | |
| Yes | 166 (70.6) | 122 (51.9) | 2.22 (1.52, 3.26) | 2.00 (1.24, 3.21) | 0.004** |
| No | 69 (29.4) | 113 (48.1) | 1 | 1 |  |
| Waist Circumference | | | | | |
| Above normal | 161 (68.5) | 121 (51.5) | 2.04 (1.40, 2.98) | 0.85 (0.37, 1.97) | 0.704 |
| Normal | 74 (31.5) | 114 (48.5) | 1 | 1 |  |
| Waist to Hip Ratio | | | | | |
| Above Normal | 160 (68.1) | 112 (47.7) | 2.34 (1.61, 3.41) | 1.84 (0.80, 4.23) | 0.150 |
| Normal | 75 (31.9) | 123 (52.3) | 1 | 1 |  |
| Family history of hypertension | | | | | |
| Yes | 120 (51.1) | 66 (28.1) | 2.67 (1.82, 3.19) | 2.13 (1.34, 3.37) | ≤ 0.001** |
| No | 115 (48.9) | 169 (71.9) | 1 | 1 |  |
| Diabetic health education | | | | | |
| No | 101 (43.0) | 65 (27.7) | 1.97 (1.34, 2.89) | 1.87 (1.18, 2.96) | 0.008** |
| Yes | 134 (57.0) | 170 (72.3) | 1 | 1 |  |
| Duration of DM | | | | | |
| >10year | 81 (34.5) | 48 (20.4) | 3.11 (1.88, 5.17) | 1.99 (1.05, 3.79) | 0.036** |
| 5-10year | 108 (46.0) | 102 (43.4) | 1.95 (1.24, 3.06) | 1.51 (0.88, 2.61) | 0.137 |
| <5year | 46 (19.6) | 85 (36.2) | 1 | 1 |  |
| Comorbidity other than hypertension | | | | | |
| Yes | 110 (46.8) | 55 (23.4) | 2.880 (1.94, 4.28) | 1.38 (0.84, 2.27) | 0.206 |
| No | 125 (53.2) | 180 (76.6) | 1 | 1 |  |
| Diabetes-related complication | | | | | |
| Yes | 57 (24.3) | 35 (14.9) | 1.82 (1.15, 2.92) | 0.79 (0.45, 1.42) | 0.444 |
| No | 178 (75.7) | 200 (85.1) | 1 | 1 |  |
| Glycemic control | | | | | |
| Poor | 109 (46.4) | 82 (34.9) | 1.61 (1.11, 2.34) | 1.57 (1.01, 2.45) | 0.046** |
| Good | 126 (53.6) | 153 (65.1) | 1 | 1 |  |

**** Indicates that variables are statistically significant at P value < 0.05**

# Discussion

The main purpose of this study was to assess determinants of hypertension among diabetic patients attending chronic follow-up clinics in Amhara region comprehensive specialized hospital. In this study diabetic patients who belong within the age group of 51-60 years were 3.33 times, 61-70 years were 3.99 times and >70 years were 2.95 times more likely to have hypertension as compared to those diabetic individuals who aged below 50 years. This finding is in line with studies conducted in Malaysia (14), Emirates (37), Benin (30), Jimma (11), Hossana (19), Debretabor (6), and as they stated that older age ( age ≥ 50years) is associated with an increased risk of having hypertension. This finding may be attributed to age-related modifications of the vascular system that results in stiffening and thickening of the layers of the artery, decreased baroreceptor sensitivity, increased responsiveness to sympathetic nervous system stimuli, alteration of renal and sodium metabolism, modification of the renin-angiotensin-aldosterone system. Those changes will finally predispose to high blood pressure (38, 39). Moreover, the possible explanation for this might also be due to easily susceptibility to pathological conditions and other DM-related complications, non-adherence with the treatments, and diabetes mellitus care. As per the finding of this study risk of having hypertension among diabetic clients aged > 70 were lower compared to diabetic clients aged 51-60 years and 61-70 years. This might be occurred due to the inclusion of the lowest proportion of cases and controls aging > 70 years compared to other age group.

In this study, the odds of having hypertension were 1.99 times higher among those individuals who live with DM for above 10 years as compared to those individuals who live with DM for less than 5 years. This finding is supported by a study conducted in Benin (30) and Adama (17). The reason might be when the duration of diabetes increases, different changes may be occurred due to chronic hyperglycemia (those changes could be damage to the vasculature, an enhanced renin-angiotensin system, increased resistance of insulin, progressive impairment of insulin secretion and sensitivity, blood vessel stiffening). Those changes finally cause endothelial suffering leading to thickening of the arterial wall and a rise in blood pressure (40, 41).

Concerning glycemic control, diabetic individuals who have poor glycemic control were 1.57 times more likely to develop hypertension than those diabetic clients with good glycemic control. This finding is in agreement with studies conducted in Debretabor (6) and Tigray, Ethiopia (18). This association is might be due to persistent hyperglycemia. Excessive level of glucose chemically binds to collagen, free amino groups of proteins, and other long-lived proteins in blood vessel walls which cause trapping of circulating low-density lipoprotein (LDL) that promotes the deposition of cholesterol in the intima, which further accelerates the formation of atheroma on the walls of arteries and subsequent hypertension. Also, hyperglycemia increases the osmolality of the extracellular fluid, causing water to shift from the intracellular to extracellular space and causing volume expansion and high BP (41-43).

The odds of having hypertension were also 1.82 times higher among diabetic individuals who had lower levels of physical activity as compared to those who have higher levels of physical activity. This finding is supported by a study conducted in Benghazi (16) and Tigray, Ethiopia (18). This association might be occurred due to the reason that exercise can normalize body mass index, improve glucose tolerance and insulin sensitivity, systemic vascular resistance, waist circumference, and able to improve insulin sensitivity. Additionally, exercise can improve lipid metabolism (e.g., increase the level of high-density lipoprotein, helps in reducing level of triglycerides, low-density lipoprotein level, and total cholesterol) which results in good glycemic control and optimal level of blood pressure (44). But if diabetic clients didn’t adhere to exercise, they might lose the advantage they have got through this physical activity,

Regarding family history, diabetic individuals who have a positive family history of hypertension were 2.13 times higher odds of having hypertension compared to those who had no family history of hypertension. This finding is in line with a study conducted in Southern Ethiopia (45) and Jordan (29). The possible reason for this might be related to different mechanisms that expose a person to high blood pressure among diabetic clients (increased renal proximal sodium reabsorption, having genetic traits related to high blood pressure such as high sodium-lithium counter-transport, elevated level of uric acid, high fasting plasma insulin concentrations and oxidative stress).

Additionally, in this study diabetic individuals who had depression were also 2 times more likely to be hypertensive than those diabetic individuals who have no depression. This finding might have occurred as a result of physiological mechanisms that may cause prolonged activation of the sympathetic nervous system and the release of inflammatory markers, catecholamines, and cortisol, which could trigger the problems of insulin resistance, which finally causes high blood pressure (46). Additionally, having depression is negatively associated with adherence to DM treatment regimen, self-care aspects (diet, medication, exercise, self-monitoring of blood glucose), medical appointments attendance, disease, and quality of life.

And also in this study, diabetic clients who didn’t attend diabetic education were 1.87 times more likely to have hypertension than their counterparts. This finding is consistent with a case-control study conducted in Tigray (18) Ethiopia. The possible reason for this might be due to diabetic education being a key factor for good blood pressure and glucose management and affecting patient health positively (22). Diabetic health education in diabetic individuals can optimize metabolic control (including self-blood glucose monitoring, adherence to dietary practice, adherence to physical activity, and medication adherence), prevent and manage complications like hypertension, improve patient and health professional’s relation and plan of care, help in adopting a more positive attitude towards the disease and relieve the symptoms of the disease or handle with emergencies and disease-related exacerbation (47). Unlike this, failure of attending diabetic education sessions is responsible for frequent re-hospitalizations, not following lifestyle modifications suggested by health care professionals, not actively engaging in self-management of diabetes, disease complications, and poor life quality.

# Strength and Limitation of the study

Using information from patients’ medical cards and using physical measurement to gather information rather than relying only on self-reported information and being a multicenter study were major strengths of this study. To the best of our knowledge, this study is also the first study to assess the effect of depression on causing hypertension. Since the study was hospital-based the finding may not be generalized to the general populations and using fasting blood glucose levels instead of HgA1C were limitations of this study. Additionally, the study might lack the power to detect the associations of hypertension with medication adherence and alcohol consumption.

# Conclusion

The burden of risk factors of hypertension among DM population is a significant public health challenge. In this study, determinants that increase the risk of hypertension among people with diabetes mellitus were increased age of respondents, physical inactivity, depression, family history of hypertension, not having a diabetic health education, longer duration of diabetes, and poor glycemic control.

# Abbreviation and Acronyms

8-MMS: 8 Item Morisky Medication Adherence Scale, AOR: Adjusted Odd Ratio, AUDIT: Alcohol Use Disorders Identification Test, BMI: Body Mass Index, CI: Confidence Interval, COR: Crude Odd Ratio, CVDs: Cardio Vascular Diseases, DM: Diabetes Mellitus, IPAQ: International Physical Activity Questionnaire, HC: Hip Circumference, HTN: Hypertension MET: Metabolic Equivalent Task, NCDs: Non-Communicable Diseases, OSLO: Oslo social support questionnaire, PDAQ: Perceived Dietary Adherence Questionnaire, PHQ 9: 9 Item Patient health Questionnaire, WC: Waist Circumference, WHO: World Health Organization, WHR: Waist to Hip Ratio

Availability of data and materials

The datasets used and/or analyzed during the current study are available from the corresponding author upon reasonable request. The datasets generated during and analyzed during the current study are not publicly available because the hospitals and the participants do not permit the researchers to share the datasets publicly.

# Acknowledgments

We are extremely thankful to Bahir Dar University, college of medicine and health science, department of adult health nursing for letting us do this research. We would also like to direct our thanks to participants, data collectors, and supervisors of this study for their cooperation and facilitation throughout the working process of this study.

**Authors' contributions**

All authors made a substantial contribution to the work reported, whether that is in the conception, execution, study design, acquisition of data, analysis and interpretation, or in all these areas; took part in revising, drafting, or critically reviewing the article; gave final approval of the version to be published; have agreed on the journal to which the article has been submitted; and agree to be accountable for all aspects of the work.

# References

1.	Sun H, Saeedi P, Karuranga S, Pinkepank M, Ogurtsova K, Duncan BB, et al. IDF diabetes atlas: Global, regional and country-level diabetes prevalence estimates for 2021 and projections for 2045. Diabetes research and clinical practice. 2022;183:109-19.

2.	Tesfaye B, Alebel A, Gebrie A, Zegeye A, Leshargie CT, Ferede A, et al. Diabetes mellitus and its association with hypertension in ethiopia: a systematic review and meta-analysis. Diabetes research and clinical practice. 2019;156:107-838.

3.	Whelton PK, Carey RM, Aronow WS, Casey DE, Collins KJ, Dennison Himmelfarb C, et al. 2017 ACC/AHA/AAPA/ABC/ACPM/AGS/APhA/ASH/ASPC/NMA/PCNA Guideline for the Prevention, Detection, Evaluation, and Management of High Blood Pressure in Adults: A Report of the American College of Cardiology/American Heart Association Task Force on Clinical Practice Guidelines. Hypertension. 2018;71(6):e13-e115.

4.	Unger T, Borghi C, Charchar F, Khan NA, Poulter NR, Prabhakaran D, et al. 2020 International Society of Hypertension global hypertension practice guidelines. Hypertension. 2020;75(6):1334-57.

5.	Ferrannini E, Cushman WC. Diabetes and hypertension: the bad companions. Lancet. 2012;380(9841):601-10.

6.	Akalu Y, Belsti Y. Hypertension and Its Associated Factors Among Type 2 Diabetes Mellitus Patients at Debre Tabor General Hospital, Northwest Ethiopia. Diabetes, Metabolic Syndrome and Obesity: Targets and Therapy. 2020;13:1621.

7.	Cho NH, Shaw JE, Karuranga S, Huang Y, da Rocha Fernandes JD, Ohlrogge AW, et al. IDF Diabetes Atlas: Global estimates of diabetes prevalence for 2017 and projections for 2045. Diabetes research and clinical practice. 2018;138:271-81.

8.	Lopez-Jaramillo P, Lopez-Lopez J, Lopez-Lopez C, Rodriguez-Alvarez MI. The goal of blood pressure in the hypertensive patient with diabetes is defined: now the challenge is go from recommendations to practice. Diabetology & Metabolic Syndrome. 2014;6(1):31.

9.	Emdin CA, Rahimi K, Neal B, Callender T, Perkovic V, Patel A. Blood pressure lowering in type 2 diabetes: a systematic review and meta-analysis. Jama. 2015;313(6):603-15.

10.	Williams R, Karuranga S, Malanda B, Saeedi P, Basit A, Besançon S, et al. Global and regional estimates and projections of diabetes-related health expenditure: Results from the International Diabetes Federation Diabetes Atlas. Diabetes research and clinical practice. 2020:108072.

11.	Abdissa D, Kene K. Prevalence and Determinants of Hypertension Among Diabetic Patients in Jimma University Medical Center, Southwest Ethiopia, 2019. Diabetes, Metabolic Syndrome and Obesity: Targets and Therapy. 2020;13:2317.

12.	Shiferaw F, Letebo M, Feleke Y, Gelibo T, Getachew T, Defar A, et al. Non-communicable diseases in Ethiopia: policy and strategy gaps in the reduction of behavioral risk factors. Ethiopian Journal of Health Development. 2019;33(4).

13.	Alqudah B, Mahmoud H, Alhusamia S, Sh A, Al L, Alawneh Z. Prevalence of hypertension among diabetic type 2 patients attending medical clinic at prince Hashem Bin. Indian J Med Res Pharm Sci. 2017;4(6):47-54.

14.	Abougalambou SSI, Abougalambou AS. A study evaluating prevalence of hypertension and risk factors affecting on blood pressure control among type 2 diabetes patients attending teaching hospital in Malaysia. Diabetes & Metabolic Syndrome: Clinical Research & Reviews. 2013;7(2):83-6.

15.	Chinedu A, Nicholas A. Hypertension prevalence and body mass index correlates among patients with diabetes mellitus in Oghara, Nigeria. The Nigerian Journal of General Practice. 2015;13(1):12.

16.	Nouh F, Omar M, Younis M. Prevalence of hypertension among diabetic patients in Benghazi: a study of associated factors. Asian Journal of Medicine and Health. 2017:1-11.

17.	Dedefo A, Galgalo A, Jarso G, Mohammed A. Prevalence of hypertension and its management pattern among type 2 diabetic patients attending, Adama Hospital Medical College, Adama. J Diabetes Metab. 2018;9(10):1-8.

18.	Mariye T, Girmay A, Tasew H, Teklay G, Ayele E, Gerensea H, et al. Determinants of hypertension among diabetic patients in Public Hospitals of the Central Zone, Tigray, Ethiopia 2018: unmatched case-control study. The Pan African Medical Journal. 2019;33.

19.	Tadesse K, Amare H, Hailemariam T, Gebremariam T. Prevalence of hypertension among patients with type 2 diabetes mellitus and its socio demographic factors in Nigist Ellen Mohamed Memorial Hospital Hosanna, Southern Ethiopia. J Diabetes Metab. 2018;9(4):792.

20.	Forde C. Scoring the international physical activity questionnaire (IPAQ). University of Dublin. 2018.

21.	Babor TF, Robaina K. The Alcohol Use Disorders Identification Test (AUDIT): A review of graded severity algorithms and national adaptations. The International Journal of Alcohol and Drug Research. 2016;5(2):17-24.

22.	FMOH. Guidelines on clinical and programmatic management of major non communicable diseases. Ethiopian Federal ministry of Health; 2016.

23.	Asaad G, Sadegian M, Lau R, Xu Y, Soria-Contreras DC, Bell RC, et al. The Reliability and Validity of the Perceived Dietary Adherence Questionnaire for People with Type 2 Diabetes. Nutrients. 2015;7(7):5484-96.

24.	Sakthong P, Chabunthom R, Charoenvisuthiwongs R. Psychometric properties of the Thai version of the 8-item Morisky Medication Adherence Scale in patients with type 2 diabetes. The Annals of pharmacotherapy. 2009;43(5):950-7.

25.	Birhanu AM, Alemu FM, Ashenafie TD, Balcha SA, Dachew BA. Depression in diabetic patients attending University of Gondar Hospital Diabetic Clinic, Northwest Ethiopia. Diabetes, metabolic syndrome and obesity : targets and therapy. 2016;9:155-62.

26.	Toobert DJ, Hampson SE, Glasgow RE. The summary of diabetes self-care activities measure: results from 7 studies and a revised scale. Diabetes care. 2000;23(7):943-50.

27.	Engidaw NA, Wubetu AD, Basha EA. Prevalence of depression and its associated factors among patients with diabetes mellitus at Tirunesh-Beijing general hospital, Addis Ababa, Ethiopia. BMC Public Health. 2020;20(1):266.

28.	Nishida C, Ko GT, Kumanyika S. Body fat distribution and noncommunicable diseases in populations: overview of the 2008 WHO Expert Consultation on Waist Circumference and Waist-Hip Ratio. European journal of clinical nutrition. 2010;64(1):2-5.

29.	Al-Azzam N, Al-Azzam S, Elsalem L, Karasneh R. Hypertension prevalence and associated factors among patients with diabetes: A retrospective cross-sectional study from Jordan. Annals of medicine and surgery (2012). 2021;61:126-31.

30.	Amoussou-Guenou D, Wanvoegbe A, Agbodandé A, Dansou A, Tchabi Y, Eyissè Y, et al. Prevalence and risk factors of hypertension in type 2 diabetics in Benin. Journal of Diabetes mellitus. 2015;5(04):227.

31.	Lee PH, Macfarlane DJ, Lam TH, Stewart SM. Validity of the international physical activity questionnaire short form (IPAQ-SF): A systematic review. International Journal of Behavioral Nutrition and Physical Activity. 2011;8(1):1-11.

32.	Feleke BE, Feleke TE, Kassahun MB, Adane WG, Fentahun N, Girma A, et al. Glycemic Control of Diabetes Mellitus Patients in Referral Hospitals of Amhara Region, Ethiopia: A Cross-Sectional Study. BioMed Research International. 2021;2021.

33.	Muche AA, Olayemi OO, Gete YK. Prevalence of gestational diabetes mellitus and associated factors among women attending antenatal care at Gondar town public health facilities, Northwest Ethiopia. BMC pregnancy and childbirth. 2019;19(1):1-13.

34.	Morisky DE, Green LW, Levine DM. Concurrent and predictive validity of a self-reported measure of medication adherence. Medical care. 1986:67-74.

35.	Gelaye B, Williams MA, Lemma S, Deyessa N, Bahretibeb Y, Shibre T, et al. Validity of the Patient Health Questionnaire-9 for depression screening and diagnosis in East Africa. Psychiatry research. 2013;210(2):653-61.

36.	Abiola T, Udofia O, Zakari M. Psychometric properties of the 3-item oslo social support scale among clinical students of Bayero University Kano, Nigeria. Malaysian Journal of Psychiatry. 2013;22(2):32-41.

37.	Mussa BM, Abduallah Y, Abusnana S. Prevalence of hypertension and obesity among emirati patients with type 2 diabetes. J Diabetes Metab. 2016;7(1):1-5.

38.	Kohn JC, Lampi MC, Reinhart-King CA. Age-related vascular stiffening: causes and consequences. Frontiers in Genetics. 2015;6(112).

39.	Head T, Daunert S, Goldschmidt-Clermont PJ. The Aging Risk and Atherosclerosis: A Fresh Look at Arterial Homeostasis. Frontiers in Genetics. 2017;8(216).

40.	Tatsumi Y, Ohkubo T. Hypertension with diabetes mellitus: significance from an epidemiological perspective for Japanese. Hypertension Research. 2017;40(9):795-806.

41.	Patel TP, Rawal K, Bagchi AK, Akolkar G, Bernardes N, Dias DDS, et al. Insulin resistance: an additional risk factor in the pathogenesis of cardiovascular disease in type 2 diabetes. Heart failure reviews. 2016;21(1):11-23.

42.	Katakami N. Mechanism of Development of Atherosclerosis and Cardiovascular Disease in Diabetes Mellitus. Journal of Atherosclerosis and Thrombosis. 2017.

43.	Wolfsdorf JI, Glaser N, Agus M, Fritsch M. ISPAD Clinical Practice Consensus Guidelines 2018: Diabetic ketoacidosis and the hyperglycemic hyperosmolar state. 2018;19 (27):155-77.

44.	Cannata F, Vadalà G, Russo F, Papalia R, Napoli N, Pozzilli P. Beneficial Effects of Physical Activity in Diabetic Patients. Journal of Functional Morphology and Kinesiology. 2020;5(3):70.

45.	Kehabtimer S. Kotiso., et al.“Prevalence and Predictors of Hypertension-Type 2 Diabetes Mellitus Comorbidity among Patients in Wachemo University Nigest Elleni Mohammed Memorial Referral Hospital, Southern Ethiopia”. EC Endocrinology and Metabolic Research. 2020;5:01-11.

46.	James K, LDominique R, Musselman, B C, Nemeroff. The relationship of depression and diabetes: Pathophysiological and treatment implications. Psychoneuroendocrinology. 2011;36(9):1276-86.

47.	Kosti M, Kanakari M. Education and diabetes mellitus. Health Science Journal. 2012;6(4):654.
